# Bioinformatic Challenges of Big Data in Non-Coding RNA Research

**DOI:** 10.3389/fgene.2012.00178

**Published:** 2012-09-11

**Authors:** Christina H. Liu, Da-Yu Wu, Jonathan D. Pollock

**Affiliations:** ^1^Division of Applied Science and Technology, National Institute of Biomedical Imaging and BioengineeringBethesda, MD, USA; ^2^Division of Basic Neuroscience and Behavior Research, National Institute on Drug Abuse, National Institutes of Health, U.S. Department of Health and Human ServicesBethesda, MD, USA

Recent technological developments have brought forth a new era of RNA research in which large sets of data are collected rapidly using the high-throughput next generation sequencing technology. Growing evidence suggests that only around 5% of nucleotides in the mammalian genomes are transcribed into protein-coding RNA, and large amount of transcripts are non-protein-coding RNA (ncRNA). During the last decade, much information has been generated from the studies of one type of ncRNA, namely microRNA (miRNA, the ncRNA of 19–25 nucleotides). miRNA modulates the expression of target genes through repression of mRNA translation or mRNA degradation. Its dysregulation has been implicated in various biological disorders and human diseases. Meanwhile, the long-non-coding RNA (lncRNA, the ncRNA that have 200 or more nucleotides) has recently emerged to catch significant attention. lncRNA is involved in chromatin modification, epigenetic regulation, transcription control, and pre- and post-translational mRNA processing. The functions of lncRNA are believed to be associated with development, imprinting, mental and psychiatric disorders, and tumor growth.

Bioinformatics is a pivotal component of this new RNA research revolution. It utilizes mathematical models and computer simulations to form, extract and analyze RNA data, and to search new ncRNA gene sequences and predict their targets. Assumptions in this computational modeling are derived from the observations that ncRNAs are produced following step-wise processes from precursors to functional end products. Based on miRNA biogenesis, criteria in searching for new miRNAs from sequencing data include that the precursors fold into a stable stem-loop structure, mature miRNAs are found on one arm of the stem, and these sequences are usually evolutionarily conserved (Lim et al., 2003). Target prediction algorithms take into considerations stability of miRNA-mRNA duplex, accessibility of secondary structure, nucleotide content in and around the putative target sites, and position of seed-complementary sites within the mRNA transcript.

Prior to the high-throughput sequencing techniques, computational programs were developed to search for new miRNAs based on attainable sequence data. These methods used one of the following approaches (Mendes et al., 2009): filter-based approaches, which identified small high-quality sets of conserved miRNA candidates; machine learning methods, which determined initial set of candidates with stem-loops structures, and target-centered approaches, which identify short conserved motifs in the 3′UTRs of protein-coding genes (Xie et al., 2005). Even though these algorithms were developed before the high-throughput sequencing era, they establish strong bases for bioinformatic analyses of big sequencing data; new ncRNAs and targets continue to be cataloged into many databases with sufficient annotations available to the public.

High-throughput sequencing techniques and deep sequencing (or RNA-Seq) have offered much improved avenue for ncRNA discovery (Lu et al., 2005), by searching genomic sequences for evidence of hairpin structures and then determine if sequencing read aligned to these structures mimic miRNA processing byproducts (Friedlander et al., 2008), or using a regularized least-squares classification algorithm to mine miRNAs from smRNA-seq data (Lu et al., 2009) to perform genome-wide multiple sequence alignments (MSAs). At the same time, through adaptation of the latest biochemical approaches to miRNA target finding, it is possible to identify miRSNPs with greater accuracy and explain the association of certain miRNA-affecting polymorphisms with disease phenotypes (Wilbert and Yeo, 2011).

Even though bioinformatic-based methods for the identification of new ncRNA and their targets have become more sophisticated and required less CPU time, there are gaps and challenges that need to be addressed to justify their biological relevancy: cross-platform validation of genomic and transcriptional sequence data, cross-algorithm validation of search engines, and development of more accurate models for ncRNA function in regard to biological environment and diseases. For example, high-throughput sequencing of small RNA results in an output file of short sequence (often termed short-reads or reads) accompanied by a quality score for each nucleotide in each sequence. Because of the high sensitivity of the technique, the “raw” data will also contain sequencing primers and contaminants which can potentially produce sequence bias that requires more sophisticated computational approaches to sieve out miRNA transcripts (Mendes et al., 2009) and cross-platform validations. There are currently at least 45 sequence formats; the most widespread data formats being those used by the major sequence database: EMBL, GenBank, SwissProt, and PIR. The lack of standardization in sequence formats not only hampers the feasibility for cross-platform comparison of existing data (Farazi et al., 2011), but also discourages the expansion of sequence data sharing for initial and value-added secondary analysis. In addition, currently available algorithms have employed different approaches dictated by the algorithm developers and may or may not be reproducible using a different approach. Cross-examination between the solutions derived from different algorithms is needed. Another complexity in ncRNA data analysis is that most of the software is primarily at a command-line level and not user-friendly to the end-users.

Computational approaches developed so far make extensive use of evolutionary conservation information either to predict ncRNA genes or ncRNA-target associations, sometimes ignoring the subtle rules presiding ncRNA biogenesis and target specificity. Thus, approaches combining high-throughput sequencing biochemical techniques and bioinformatic analyses that emphasizes the synergy of genome-wide approaches are essential (Mendes et al., 2009). Furthermore, most lncRNA are under lower sequence constraints than protein-coding genes and lack conserved secondary structures like the pre-miRNAs, making it hard to predict computationally. In addition, since complex diseases can be affected by a number of ncRNAs rather than a single ncRNA, and ncRNA often operates in highly complex regulatory networks (Kargul and Laurent, 2011), it is a multi-dimensional challenge to identify ncRNA interactions at a system-wide level, and analyze the roles of ncRNA in disease and disorders in the ncRNA–ncRNA synergistic network (Xu et al., 2011). Lastly, careful interpretations of data with molecular validations are critical for ensuring acceptance of bioinformatic methods in the ncRNA research community. With knowledge gained from bioinformatic analyses of exponentially increasing massive ncRNA data, many issues remain to be addressed on the functional significance and how genetic variations of ncRNA plays important roles in disease processes.
